# Supplementary material for: A comparative study between state‐of‐the‐art MRI deidentification and AnonyMI, a new method combining re‐identification risk reduction and geometrical preservation
Source: Hum Brain Mapp. 2021 Sep 14;42(17):5523–34. doi: 10.1002/hbm.25639 (PMC8559469; doi:10.1002/hbm.25639)
Supplement: Supplementary file 1 — FIGURE S1 Description and example of the Image processing steps applied for the creation of the behavioral experiments' stimuli. TABLE S1 Detailed results of the analysis of Experiment 1. (a) Mixed effects model. (b) Estimated marginal means post‐hoc contrasts with Tukey correction for multiple comparisons. TABLE S2 Detailed results of the analysis of Experiment 1. (a) Mixed effects model. (b) Estimated marginal means post‐hoc contrasts with Tukey correction for multiple comparisons. TABLE S3. Detailed results of the analysis of the Hausdorff Distance. Letters indicate surfaces being compared. Top sub‐tables correspond to descriptive statistics and lower sub‐tables correspond to p values of Wilcoxon signed rank tests with Holm–Bonferroni correction for multiple comparisons. TABLE S4. Detailed results of the source localization analysis. Mixed effects model. [file HBM-42-5523-s001.pdf]

Supplementary Materials

Title

A comparative study between state-of-the-art MRI deidentification and Anony-MI, a new method combining re-identification risk reduction and geometrical preservation

Abstract

Deidentifying MRIs constitutes an imperative challenge, as it aims at precluding the possibility of re-identification of a research subject or patient, but at the same time it should preserve as much geometrical information as possible, in order to maximize data reusability and to facilitate interoperability. Although several anonymization methods exist, no comprehensive and comparative evaluation of deidentification performance has been carried out across them. Moreover, the possible ways these methods can compromise subsequent analysis has not been exhaustively tested. To tackle these issues, we developed AnonyMI, a novel MRI deidentification method, implemented as a user-friendly 3D Slicer plugin-in, which aims at providing a balance between identity protection and geometrical preservation. To test these features, we performed two series of analyses on which we compared AnonyMI to other two state-of-the-art methods, to evaluate, at the same time, how efficient they are on deidentifying MRIs and how much they affect subsequent analyses, with particular emphasis on source localization procedures. Our results show that all three methods significantly reduce the re-identification risk but AnonyMI provides the best geometrical conservation. Notably, it also offers several technical advantages such as a user-friendly interface, multiple input-output capabilities, the possibility of being tailored to specific needs, batch processing and efficient visualization for quality assurance.*_

Supplementary Figures

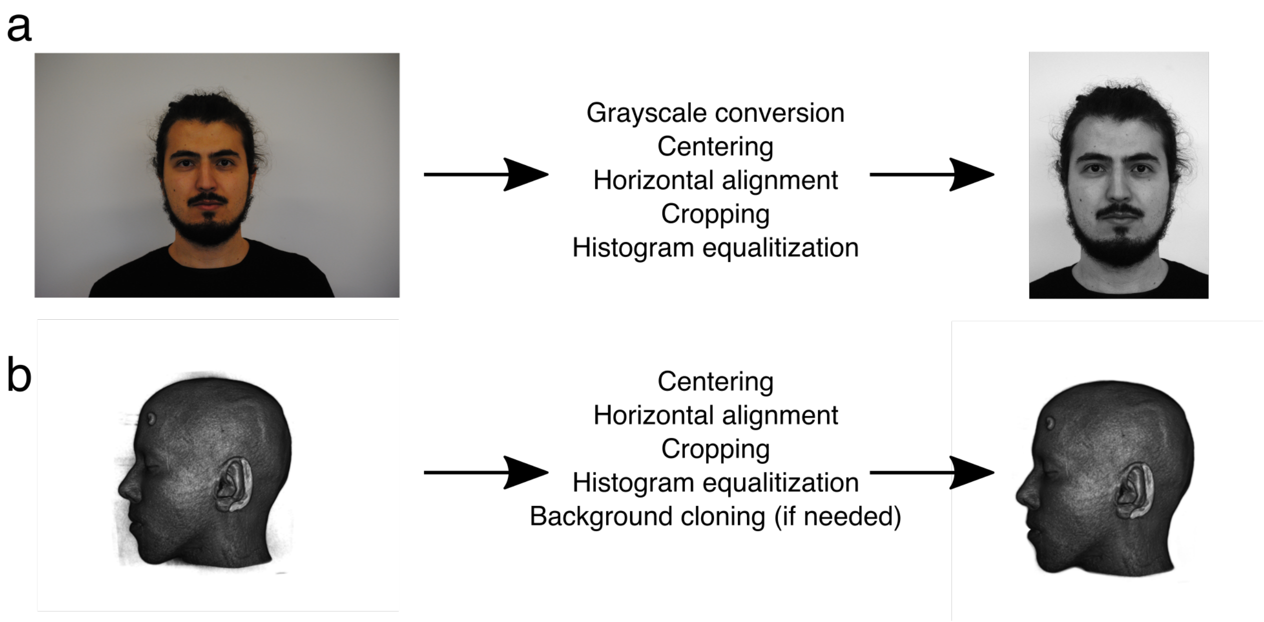

Supplementary Figure 1. Description and example of the Image processing steps applied for the creation of the behavioral experiments' stimuli.

Supplementary Tables

```
a) MIXED EFFECTS MODEL
     AIC      BIC   logLik deviance df.resid 
  5506.8   5532.2  -2749.4   5498.8     4181 

Scaled residuals: 
    Min      1Q  Median      3Q     Max 
-0.8370 -0.7814 -0.7070  1.2497  1.4596 

Random effects:
 Groups      Name        Variance Std.Dev.
 participant (Intercept) 0.005228 0.0723  
Number of obs: 4185, groups:  participant, 31

Fixed effects:
              Estimate Std. Error z value Pr(>|z|)    
(Intercept)   -0.69402    0.05832 -11.901  < 2e-16 ***
methodanonymi  0.18216    0.07932   2.296  0.02166 *  
methodmf       0.28803    0.07887   3.652  0.00026 ***
---
Signif. codes:  0 ‘***’ 0.001 ‘**’ 0.01 ‘*’ 0.05 ‘.’ 0.1 ‘ ’ 1

Correlation of Fixed Effects:
            (Intr) mthdnn
methodannym -0.698       
methodmf    -0.702  0.516

b) ESTIMATED MARGINAL MEANS
 method  emmean     SE  df asymp.LCL asymp.UCL
 deface  -0.694 0.0583 Inf    -0.808    -0.580
 anonymi -0.512 0.0569 Inf    -0.623    -0.400
 mf      -0.406 0.0562 Inf    -0.516    -0.296

Results are given on the logit (not the response) scale. 
Confidence level used: 0.95 
 contrast         estimate     SE  df z.ratio p.value
 deface - anonymi   -0.182 0.0793 Inf -2.296  0.0563 
 deface - mf        -0.288 0.0789 Inf -3.652  0.0008 
 anonymi - mf       -0.106 0.0778 Inf -1.361  0.3617 
```

Supplementary Table 1. Detailed results of the analysis of Experiment 1. a) Mixed effects model. b) Estimated marginal means post-hoc contrasts with Tukey correction for multiple comparisons.

```
a) MIXED EFFECTS MODEL
     AIC      BIC   logLik deviance df.resid
  2852.3   2881.0  -1421.2   2842.3     2299

Scaled residuals:
    Min      1Q  Median      3Q     Max
-0.9568 -0.6602 -0.6042  1.1379  1.9102

Random effects:
 Groups      Name        Variance Std.Dev.
 participant (Intercept) 0.02011  0.1418
Number of obs: 2304, groups:  participant, 24

Fixed effects:
              Estimate Std. Error z value Pr(>|z|)
(Intercept)   -0.23134    0.08894  -2.601   0.0093 **
methodanonymi -0.64334    0.12433  -5.175 2.28e-07 ***
methodmf      -0.61833    0.12399  -4.987 6.14e-07 ***
methoddeface  -0.86303    0.12780  -6.753 1.45e-11 ***
---
Signif. codes:  0 '***' 0.001 '**' 0.01 '*' 0.05 '.' 0.1 ' ' 1

Correlation of Fixed Effects:
            (Intr) mthdnn mthdmf
methodannym -0.639
methodmf    -0.641  0.459
methoddefac -0.622  0.446  0.447

b) ESTIMATED MARGINAL MEANS
 method  emmean     SE  df asymp.LCL asymp.UCL
 orig    -0.231 0.0889 Inf    -0.406    -0.057
 anonymi -0.875 0.0961 Inf    -1.063    -0.686
 mf      -0.850 0.0956 Inf    -1.037    -0.662
 deface  -1.094 0.1005 Inf    -1.291    -0.897

Results are given on the logit (not the response) scale.
Confidence level used: 0.95
 contrast         estimate    SE  df z.ratio p.value
 orig - anonymi      0.643 0.124 Inf  5.175  <.0001
 orig - mf           0.618 0.124 Inf  4.987  <.0001
 orig - deface       0.863 0.128 Inf  6.753  <.0001
 anonymi - mf       -0.025 0.129 Inf -0.194  0.9974
 anonymi - deface    0.220 0.133 Inf  1.655  0.3480
 mf - deface         0.245 0.132 Inf  1.847  0.2511
```

Supplementary Table 2. Detailed results of the analysis of Experiment 1. a) Mixed effects model. b) Estimated marginal means post-hoc contrasts with Tukey correction for multiple comparisons.

```
a) BRAIN
  method  median  mean    sd
1 anonymi   5.58  5.94  2.12
2 mf        5.90  8.10  7.86
3 deface    6.11  6.84  5.20

       anonymi mf
mf     0.83    -
deface 0.87    0.87

b) INNER SKULL
  method  median  mean    sd
1 anonymi   4.50  5.04  2.19
2 mf        4.99  7.00  7.28
3 deface    5.09  5.96  5.44

       anonymi mf
mf     0.88    -
deface 1.00    1.00

c) OUTER SKULL
  method  median  mean    sd
1 anonymi   4.55  4.69  1.89
2 mf        4.47  6.00  5.05
3 deface   49.0  49.3   3.68

       anonymi mf
mf     0.25    -
deface 8.5e-14 8.5e-14

d) OUTER SKIN
  method  median  mean    sd
1 anonymi   5.26  5.42  2.23
2 mf        5.68  7.32  5.13
3 deface   49.6  50.2   3.77

       anonymi mf
mf     0.0012  -
deface 8.5e-14 8.5e-14
```

Supplementary Table 3. Detailed results of the analysis of the Hausdorff Distance. Letters indicate surfaces being compared. Top sub-tables correspond to descriptive statistics and lower sub-tables correspond to p-values of Wilcoxon signed rank tests with Holm-Bonferroni correction for multiple comparisons.

```
Scaled residuals:
    Min      1Q  Median      3Q     Max 
-3.7373 -0.6829 -0.0868  0.4017  4.5784 

Random effects:
 Groups   Name          Variance Std.Dev. Corr             
 subj     (Intercept)   0.115507 0.33986                   
          anon_manonymi 0.007997 0.08942  -0.84            
          anon_mmf      0.029306 0.17119  -0.47  0.60      
          anon_mdefaced 0.008449 0.09192  -0.69  0.80  0.50
 Residual               0.202746 0.45027                   
Number of obs: 3728, groups:  subj, 7

Fixed effects:
              Estimate Std. Error      df t value Pr(>|t|)    
(Intercept)    1.58672    0.12944 6.03326  12.258 1.72e-05 ***
anon_manonymi  0.14349    0.04022 5.95712   3.568  0.01196 *  
anon_mmf       0.18979    0.06858 5.97269   2.767  0.03269 *  
anon_mdefaced  0.17626    0.04121 5.73197   4.277  0.00579 ** 
---
Signif. codes:  0 ‘***’ 0.001 ‘**’ 0.01 ‘*’ 0.05 ‘.’ 0.1 ‘ ’ 1

Correlation of Fixed Effects:
            (Intr) ann_mn ann_mm
anon_mannym -0.751              
anon_mmf    -0.468  0.564       
anon_mdefcd -0.627  0.715  0.487
```

Supplementary Table 4. Detailed results of the source localization analysis. Mixed effects model.
